# Pharmacokinetics and Tissue Distribution Study of Praeruptorin D from Radix Peucedani in Rats by High-Performance Liquid Chromatography (HPLC)

**DOI:** 10.3390/ijms13079129

**Published:** 2012-07-20

**Authors:** Taigang Liang, Wenyan Yue, Xue Du, Luhui Ren, Qingshan Li

**Affiliations:** School of Pharmaceutical Science, Shanxi Medical University, No 56, Xinjian Nan Road, Taiyuan 030001, Shanxi, China; E-Mails: ltaigang@gmail.com (T.L.); yuewenyanhappy@163.com (W.Y.); duxue_1988@163.com (X.D.); renluhui@126.com (L.R.)

**Keywords:** praeruptorin D, Radix Peucedani, HPLC, pharmacokinetics, tissue distribution

## Abstract

Praeruptorin D (PD), a major pyranocoumarin isolated from Radix Peucedani, exhibited antitumor and anti-inflammatory activities. The aim of this study was to investigate the pharmacokinetics and tissue distribution of PD in rats following intravenous (i.v.) administration. The levels of PD in plasma and tissues were measured by a simple and sensitive reversed-phase high-performance liquid chromatography (HPLC) method. The biosamples were treated by liquid-liquid extraction (LLE) with methyl *tert*-butyl ether (MTBE) and osthole was used as the internal standard (IS). The chromatographic separation was accomplished on a reversed-phase C_18_ column using methanol-water (75:25, *v*/*v*) as mobile phase at a flow rate of 0.8 mL/min and ultraviolet detection wave length was set at 323 nm. The results demonstrate that this method has excellent specificity, linearity, precision, accuracy and recovery. The pharmacokinetic study found that PD fitted well into a two-compartment model with a fast distribution phase and a relative slow elimination phase. Tissue distribution showed that the highest concentration was observed in the lung, followed by heart, liver and kidney. Furthermore, PD can also be detected in the brain, which indicated that PD could cross the blood-brain barrier after i.v. administration.

## 1. Introduction

**Figure 1:**
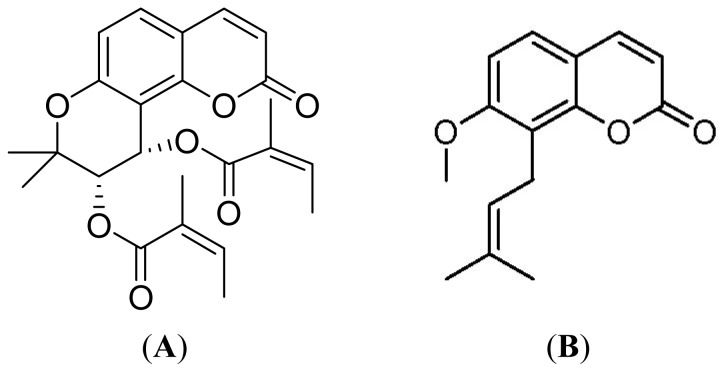
Chemical structures of praeruptorin D (PD) (**A**) and Osthole (IS) (**B**).

Radix Peucedani (Baihua Qianhu in Chinese), the dried roots of *Peucedanum praeruptorum* DUNN (Umbelliferae), is a well-known traditional Chinese medicinal herb which was officially listed in the Chinese Pharmacopoeia [1]. It has been widely used in China for centuries in the treatment of coughs with thick sputum and dyspnea, upper respiratory infections, and nonproductive cough [2]. Phytochemical investigations reveal that coumarins are widely distributed in this plant [3–6]. Among them, angular-type pyranocoumarins (seselins) were regarded as the principal components responsible for the main pharmacological activities. Isolation of pyranocoumarins (praeruptorins A, B, C and D) from this herb was first reported by Chen *et al*. [3]. Praeruptorin A (PA) was found to be racemic to praeruptorin C (PC), and praeruptorin B (PB) to praeruptorin D (PD, Figure 1A), respectively. Previous studies indicated that PA exhibited a variety of biological activities including anticancer [7–9], vasodilatation [10], reversing multidrug resistance (MDR) [11,12] and calcium antagonistic action [13,14], *etc*. Moreover, the pharmacokinetics, tissue distribution, metabolism and excretion of PA have been well documented [15–19]. On the other hand, PD, as a PA analogue, has also drawn extensive attention in recent years since it was found to possess some other specific pharmacological effects such as inhibiting tumor promoter induced phenomenon *in vitro* [20] and potent anti-inflammatory action in addition to the above-mentioned similar activities [21].

Analytical techniques applied to the quantification of PD from herbal medicines included thin-layer chromatography (TLC) [1] and high-performance liquid chromatography (HPLC) with UV detection [22]. Recently, a liquid chromatography coupled with mass spectrometry (HPLC-MS/MS) method was reported to investigate the metabolism of PD *in vitro* [23]. With the growing significance of a potential beneficial role of PD in human health, there is an increasing demand for investigating its pharmacokinetics *in vivo*. However, to our knowledge, there has been no research on the pharmacokinetics and tissue distribution in rat after administration of PD. Nowadays, the HPLC-UV method has been commonly used to determine drug contents in complex biological samples. In the present paper, a rapid and sensitive HPLC-UV method was developed and validated to determine PD in rat plasma and tissue homogenates. The method was successfully applied to pharmacokinetics and tissue distribution study after i.v. administration of PD to healthy rats.

## 2. Results and Discussion

### 2.1. Preparation of Plasma and Tissue Samples

Biosample preparation was performed using a liquid-liquid extraction (LLE) and different extraction solvents such as ethyl acetate, chloroform, hexane, diethyl ether, and methyl *tert*-butyl ether (MTBE) were investigated. Finally, MTBE was found to be optimal, because it can produce clean chromatograms for plasma and tissues and yielded the highest recovery for the analytes.

### 2.2. Method Validation

#### 2.2.1. Specificity

**Figure 2:**
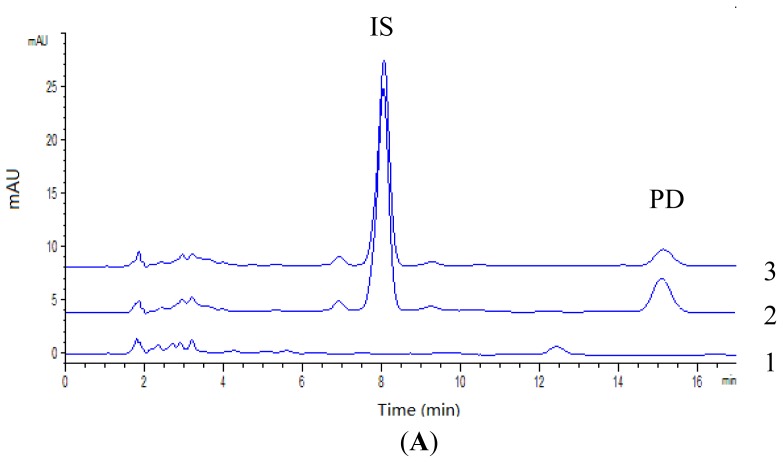

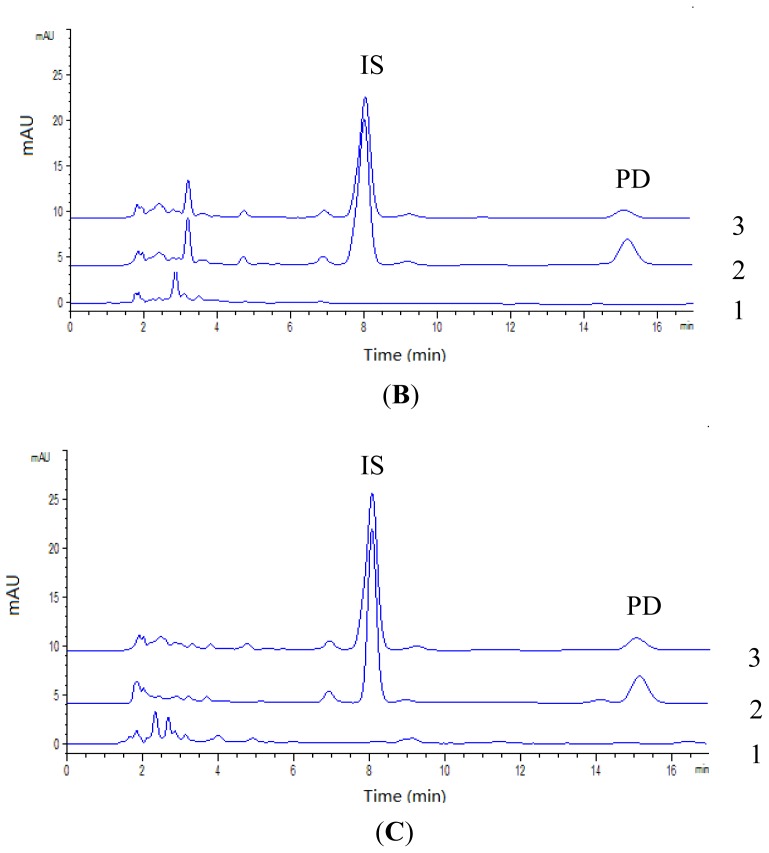
Representative chromatograms of plasma (**A**), liver (**B**) and lung (**C**). 1. blank plasma or tissues (liver and lung); 2. blank plasma or tissues (liver and lung) spiked with PD and IS; 3. plasma or tissues (liver and lung) sample at 60 min following i.v. administration of PD at a single dose of 10 mg/kg.

The representative chromatograms for determination of PD in plasma and tissues (liver and lung were chosen as representative tissues) are shown in Figure 2A–C. The retention time of IS was about 7.9 min and PD was 15.2 min. It was indicated that analytes were well separated and no interferences were detected from endogenous substances or metabolites.

#### 2.2.2. Linearity of Calibration Curve and Lower Limit of Quantification

The calibration curves showed good linearity over the concentration range of 0.0512–52.2 μg/mL in rat plasma and tissue homogenates with a correlation coefficient (*R*^2^) larger than 0.997. Typical linear regression equations, correlation coefficients and linear ranges of PD in plasma and each tissue were listed in Table 1. The current assay offered an LLOQ of 0.0512 μg/mL in plasma and tissue samples. The limits were sufficient for studies of pharmacokinetics and tissue distribution following a single i.v. administration of PD.

#### 2.2.3. Precision and Accuracy

The precision and accuracy of the method were summarized in Table 2. For all the samples spiked with analytes at three concentration levels, the RSD% of both intra-day and inter-day precision was below 11.25%, and the accuracy was within the range of −11.03 to 10.19%. The results demonstrated that the method is accurate and reproducible for determination of PD in rat plasma and tissues.

#### 2.2.4. Recovery and Stability

The extraction recoveries of PD ranged from 82.07% to 88.63% in plasma and tissue samples (Table 2), while the recovery of IS was above 80% (data not shown). These data indicated the biosample preparation procedure was satisfied and can achieve the acceptable extraction recovery.

The stability tests were designed to cover the anticipated conditions that the samples may experience. The results are summarized in Table 3, which showed that PD remained stable in above three conditions.

### 2.3. Pharmacokinetics of PD in Rats

**Figure 3:**
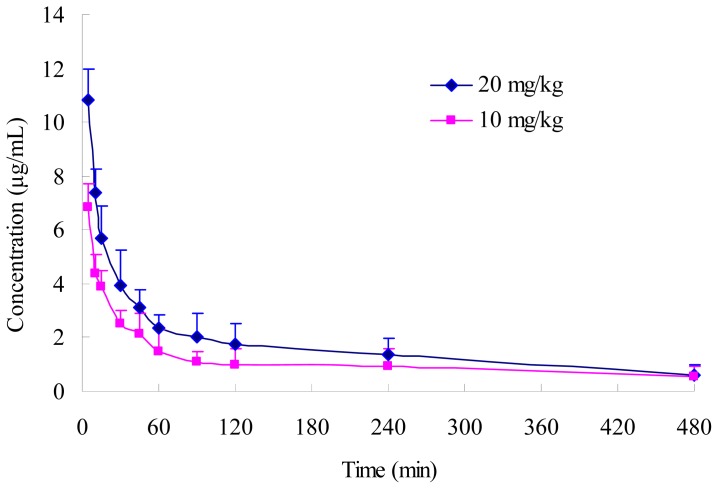
Plasma concentration-time profiles of PD following i.v. administration in rats at doses of 10 and 20 mg/kg (*n* = 6).

The mean plasma concentration-time profiles of PD in rats following i.v. administration at doses of 10 and 20 mg/kg are shown in Figure 3 and the corresponding pharmacokinetic parameters are summarized in Table 4. It was found that the data were best fitted with a two-compartment model. PD had a fast distribution phase (t_1/2α_, 0.119–0.130 h) followed by a relative slow elimination phase (*t*_1/2β_, 2.408–2.640 h) and could be detected until 480 min post dosing using the analytical method described above. A dose proportionality study indicated that there is good correlation between AUC and dose. Significant difference in AUC between the two doses was observed (*p* < 0.05). On the other hand, there was no significant difference in systemic clearance (CLs) at two dose levels, suggesting that PD may have linear pharmacokinetic characteristics in rats within the dose ranges tested [24].

Furthermore, we also tried to conduct pharmacokinetics study of PD given to rats by oral route. However, the concentration of PD in plasma was too low to be detected even the oral dose was up to 200 mg/kg, which indicated that PD might have a poor absorption from gastrointestinal tract in rat.

### 2.4. Tissue Distribution Study

**Figure 4:**
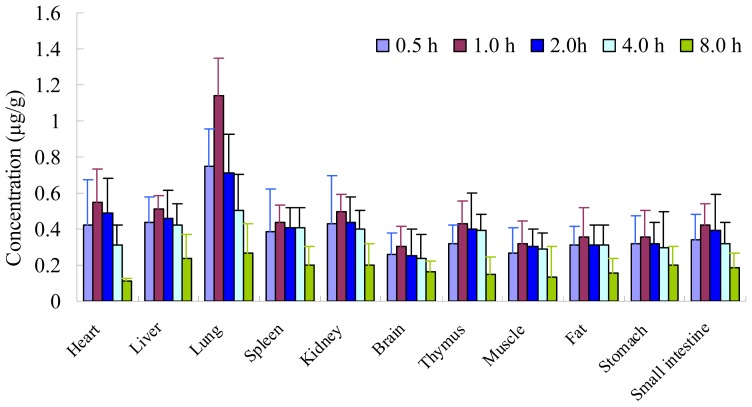
Tissue distribution of PD at times 0.5, 1.0, 2.0, 4.0 and 8.0 h after i.v. injection of 20 mg/kg in rats (*n* = 6).

The tissue distribution of PD after i.v. administration of 20 mg/kg in rats at 0.5, 1.0, 2.0, 4.0 and 8.0 h was presented in Figure 4. At 1.0 h after administration of PD to rats, the highest level of PD was observed in all collected tissues. Even at 8.0 h, PD was still detectable in all tissues, but obvious downward trend. The results indicated that the PD underwent a rapid and wide distribution in tissues within the time course examined. The highest tissue concentrations were found in the lung, followed by heart, liver and kidney, which implied that the distribution of PD depended on the blood flow or perfusion rate of the organ. The high affinity in lung of PD confirms that PD was a major bioactive component in Radix Peucedani which has good curative effect on respiratory diseases in traditional Chinese medicine. In addition, PD could also be detected in brain homogenate, suggesting that PD can efficiently cross the blood-brain barrier.

## 3. Experimental Section

### 3.1. Materials and Reagents

PD was isolated from ethanol extract of the roots of Radix Peucedani as follows: The dried roots (1 kg) were powdered and extracted with 95% ethanol under reflux for three times (3 × 8 L). The combined extract was evaporated under vacuum at 45 °C. Afterwards, the residue was dissolved in a mixture of H_2_O-ethyl acetate (1:1) and partitioned, the resulting ethyl acetate fraction was separated by column chomatography (CC) on silica gel using petroleum ether-ethyl acetate (7:1) to give PD (2.8 g). The structure was identified by comparison of its physico-chemical (melting point and optical rotation) and spectroscopic (MS, UV, ^1^H NMR, ^13^C NMR and IR) data [3]. Its purity was determined to be over 98.0% by HPLC. Osthole (Figure 1B), the internal standard (IS), was obtained from the National Institute for the Control of Pharmaceutical and Biological Products (Beijing, China). HPLC grade methanol was purchased from Tianjin Concord Technology Co. Ltd. (Tianjin, China). Deionized water was purified by Milli-Q system (Millipore, Bedford, MA, USA). Other chemicals used were of analytical grade.

### 3.2. Animals

Male Sprague-Dawley (SD) rats (10–12 weeks old, weighing 200–230 g) were obtained from the department of Laboratory Animal Science of Shanxi Medical University (Taiyuan, China). Animal welfare and experimental procedures were strictly in accordance with the related ethics regulations of Shanxi Medical University. All animals were kept in an environmentally controlled breeding room (temperature maintained at about 25 ± 2 °C and with a 12 h light/12 h dark cycle) for at least one week before starting the experiments and fed with standard laboratory food and water *ad libitum*. Prior to each experiment, the rats were fasted for 12 h with free access to water.

### 3.3. Instrumentation and Chromatographic Conditions

The HPLC analysis was carried out on an Agilent 1200 series liquid chromatographic system (Agilent Technologies, Santa Clara, CA, USA) equipped with G1311A quaternary pump, G1316A thermostatted column compartment, G1322A vacuum degasser, G1329A auto-sampler, and G1315B diode array detector (DAD). Data acquisition was controlled by an Agilent ChemStation B 3.0 software. Chromatographic separation was accomplished on a Diamonsil C_18_ (4.6 × 150 mm I.D., 5 μm particle size) analytical column (Dikma Technologies Co. Ltd, Beijing, China). The mobile phase was methanol-water (75:25, *v*/*v*) at a flow rate of 0.8 mL/min. Chromatograms were monitored at 323 nm and the column temperature was maintained at 25 °C.

### 3.4. Calibration Standards and Quality Control (QC) Samples Preparation

Stock solution of PD was prepared in methanol to give a final concentration of 1.024 mg/mL. A series of working solutions were obtained by diluting the PD stock solution with methanol. The stock solution of IS (1.0 mg/mL) was also diluted to concentration 10 μg/mL with methanol as working solution. All solutions were stored at 4 °C until used. Calibration standards of PD were prepared by spiking the appropriate amount of the working solutions into 100 μL drug-free rat plasma or tissue homogenates. The final concentrations of calibration standard samples were 0.0512, 0.256, 0.64, 3.2, 12.8 and 51.2 μg/mL.

Quality control (QC) samples were prepared at low, medium and high concentrations of 0.0512, 3.2, 51.2 μg/mL for plasma and different tissue homogenates in the same manner as the calibration standards.

### 3.5. General Procedure of Sample Preparation for PD Analysis in Plasma and Tissue Samples

A liquid-liquid extraction (LLE) of PD in biosamples was performed prior to HPLC analysis. Briefly, 50 μL of IS working solution (10 μg/mL) was added to 100 μL of plasma or tissue samples in glass centrifuge tubes. The mixture was extracted twice with 3 mL MTBE by vortexing for 5 min. After centrifugation at 10,000 rpm for 5 min, the upper organic layer was transferred to a clean tube and evaporated to dryness under a gentle stream of nitrogen at 40 °C. The residue was reconstituted in 100 μL of methanol and centrifuged at 10,000 rpm for 5 min. An aliquot (20 μL) of supernatant was injected onto the HPLC system.

### 3.6. Method Validation

#### 3.6.1. Specificity

Specificity was assessed by analyzing blank plasma and tissue homogenate samples, blank plasma and tissue homogenate samples spiked with PD, and rat plasma and tissue samples after i.v. administration of PD.

#### 3.6.2. Calibration Curves and Lower Limit of Quantification

Calibration standard was prepared as described above in triplicate and analyzed on three consequent days. Calibration curves were constructed by plotting the peak area ratio of PD to IS against the concentration of PD using 1/X^2^ as weighting factor.

The lower limit of quantification (LLOQ) was defined as the lowest concentration of analyte in a sample which provided a peak area with a signal-to-noise ratio higher than 10.

#### 3.6.3. Precision and Accuracy

Intra-day precision and accuracy were evaluated by analysis of the three QC samples with six determinations per concentration at the same day, whilst the inter-day precision and accuracy were measured over three consecutive days. The precision was defined as the relative standard deviation (RSD%), while accuracy was determined by calculating the percentage deviation observed in the analysis of QC samples and expressed by relative error (RE%). The accepted criteria for the data were that the precision and accuracy should not exceed 15%, except at the LLOQ where it should not exceed 20%.

#### 3.6.4. Extraction Recovery and Stability

The extraction recoveries of PD were determined at low, medium and high level of QC samples. Recoveries were calculated by comparing the observed peak area ratios in biosamples to those non-processed standard solutions at the same concentrations. The recovery of IS was determined in the same way at the concentration of 10 μg/mL.

The stability of PD in plasma and tissue was determined under different storage or handling conditions. Short-term stability was assessed by analyzing QC samples kept at ambient temperature for 8 h. Freeze-thaw stability was evaluated at three consecutive freeze-thaw cycles. Long-term stability was studied by assaying samples following a period of 2 weeks of storage at −70 °C.

### 3.7. *In Vivo* Pharmacokinetic Study

Twelve rats were randomly assigned to two groups for pharmacokinetic investigation (*n* = 6 per group). PD was administered by i.v. injection via the lateral tail vein at the dose of 10 and 20 mg/kg, respectively. At the time points of 0 (pre-dose), 5, 15, 30, 45, 60, 90, 120, 240 and 480 min post injection, blood samples (0.5 mL) were collected in heparinized tubes from the orbital vein, and then centrifuged at 10,000 rpm for 5 min to obtain plasma. The plasma was stored at −70 °C prior to analysis by HPLC.

### 3.8. Tissue Distribution Study

For tissue distribution study, thirty rats were divided into five groups (*n* = 6 per group) randomly and PD was administered intravenously through the tail vein at a dose of 20 mg/kg. After injection, the rats were sacrificed at 0.5, 1.0, 2.0, 4.0 and 8.0 h following administration, and the tissue specimens including lung, liver, heart, spleen, stomach, small intestine, brain, thymus, muscle, fat and kidney were collected. Tissue samples were rinsed in saline and blotted dry with filter paper, and then weighed for wet weight and homogenized in ice-cold physiological saline solution (500 mg/mL). The obtained tissue homogenates were stored at −70 °C until analysis performed using the procedure described above.

### 3.9. Statistical Analysis

The pharmacokinetic parameters were calculated using the 3P97 software (Chinese Pharmacology Society: Beijing, China, 1987). An appropriate pharmacokinetic model was chosen based on the lowest Akaike’s information criterion (AIC) value, lowest weighted squared residuals, lowest standard errors of the fitting parameters, and dispersion of the residual under equal weight scheme [25–27]. All the data were expressed as the mean ± standard deviation and the levels of statistical significance were assessed using Student *t* test.

## 4. Conclusions

In conclusion, we have developed a simple, rapid and sensitive method for the quantitative determination of PD in biological samples including plasma and tissues. The achieved pharmacokinetics and tissue distribution results may be useful for further study of the bioactive mechanism of PD.

## Figures and Tables

**Table 1:** The calibration curves, coefficients and linear ranges of PD in plasma and tissue samples.

Biosamples	Calibration curves	*R*^2^	Linear range (μg/mL)
Plasma	*Y* = 0.0876*X* + 0.0016	0.9991	0.0512–51.2
Lung	*Y* = 0.1074*X* + 0.0054	0.9999	0.0512–51.2
Liver	*Y* = 0.1426*X* + 0.0812	0.9984	0.0512–51.2
Heart	*Y* = 0.1314*X* + 0.0094	0.9989	0.0512–51.2
Spleen	*Y* = 0.1381*X* + 0.0017	0.9993	0.0512–51.2
Stomach	*Y* = 0.1318*X* + 0.0142	0.9981	0.0512–51.2
Small intestine	*Y* = 0.1315*X* + 0.0072	0.9973	0.0512–51.2
Brain	*Y* = 0.1417*X* + 0.0114	0.9988	0.0512–51.2
Thymus	*Y* = 0.1381*X* + 0.0020	0.9994	0.0512–51.2
Muscle	*Y* = 0.1493*X* + 0.0147	0.9982	0.0512–51.2
Fat	*Y* = 0.1517*X* + 0.0039	0.9979	0.0512–51.2
Kidney	*Y* = 0.1359*X* + 0.0018	0.9991	0.0512–51.2

**Table 2:** Accuracy, precision and recovery of the method used for determination of PD in rat plasma and tissue samples (*n* = 6).

Biosamples	Concentration (μg/mL)	Intra-day		Inter-day		Recovery
		
		Precision (RSD%)	Accuracy (RE%)	Precision (RSD%)	Accuracy (RE%)	Mean ± SD (%)
Plasma	0.0512	8.80	9.79	11.25	−10.56	84.00 ± 5.88
	3.2	5.43	−8.94	6.20	8.01	83.12 ± 4.06
	51.2	2.91	−3.62	4.07	5.98	88.63 ± 6.75
Liver	0.0512	7.29	−8.51	9.62	−11.03	86.56 ± 5.31
	3.2	8.47	−8.48	9.97	−5.44	84.51 ± 5.64
	51.2	5.56	6.33	5.56	3.07	83.63 ± 6.93
Lung	0.0512	6.90	9.14	5.94	10.19	84.35 ± 5.20
	3.2	6.62	−5.75	8.90	7.85	85.12 ± 4.17
	51.2	5.88	6.87	10.52	7.19	82.07 ± 6.42

**Table 3:** Stability of PD in plasma, liver and lung samples of rats (*n* = 6).

Biosamples	Concentration (μg/mL)	Accuracy (RE%)		

		Short-term stability	Freeze-thaw stability	Long term stability
Plasma	0.0512	−11.76	10.28	9.30
	3.2	−5.22	−8.75	−7.04
	51.2	4.43	−7.11	8.89
Liver	0.0512	−8.05	5.29	−8.57
	3.2	7.57	−6.45	−10.81
	51.2	5.81	7.76	6.06
Lung	0.0512	−11.35	9.03	−7.15
	3.2	−9.76	7.05	6.92
	51.2	8.13	5.02	−4.25

**Table 4:** The main pharmacokinetic parameters of PD after i.v. administration (*n* = 6) *t*_1/2α_: distribution phase half-life; *t*_1/2β_:elimination phase; AUC_0-∞_: Area under the concentration-time curve from zero up to infinite time; CLs: systemic clearance.

Parameter	Unit	Dose	

		10 mg/kg	20 mg/kg
t_1/2α_	h	0.119 ± 0.036	0.130 ± 0.045
t_1/2β_	h	2.408 ± 0.409	2.640 ± 0.612
AUC_0-∞_	mg·h/L	18.145 ± 6.265	40.790 ± 11.746 *
CLs	L/h/kg	0.576 ± 0.193	0.513 ± 0.185

* *p* < 0.05, compared with the value of 10 mg/kg dose.
